# Suppression of Colorectal Cancer Liver Metastasis by Apolipoprotein(a) Kringle V in a Nude Mouse Model through the Induction of Apoptosis in Tumor-Associated Endothelial Cells

**DOI:** 10.1371/journal.pone.0093794

**Published:** 2014-04-03

**Authors:** Jin-Hyung Ahn, Hyun-Kyung Yu, Ho-Jeong Lee, Soon Won Hong, Sun Jin Kim, Jang-Seong Kim

**Affiliations:** 1 Cancer Biology Team, Mogam Biotechnology Research Institute, Yongin, Republic of Korea; 2 Department of Pathology, Gangnam Sevrance Hospital, Yonsei University, Seoul, Republic of Korea; 3 Department of Cancer Biology, The University of Texas MD Anderson Cancer Center, Houston, Texas, United States of America; 4 Research Center of Integrative Cellulomics, Korea Research Institute of Bioscience and Biotechnology, Daejeon, Republic of Korea; Seoul National University, Republic of Korea

## Abstract

The formation of liver metastases in colorectal cancer patients is the primary cause of patient death. Current therapies directed at liver metastasis from colorectal cancer have had minimal impact on patient outcomes. Therefore, the development of alternative treatment strategies for liver metastasis is needed. In the present study, we demonstrated that recombinant human apolipoprotein(a) kringle V, also known as rhLK8, induced the apoptotic turnover of endothelial cells *in vitro* through the mitochondrial apoptosis pathway. The interaction of rhLK8 with glucose-regulated protein 78 (GRP78) may be involved in the induction of apoptosis because the inhibition of GRP78 by GRP78-specific antibodies or siRNA knockdown inhibited the rhLK8-mediated apoptosis of human umbilical vein endothelial cells *in vitro*. Next, to evaluate the effects of rhLK8 on angiogenesis and metastasis, an experimental model of liver metastasis was established by injecting a human colorectal cancer cell line, LS174T, into the spleens of BALB/c nude mice. The systemic administration of rhLK8 significantly suppressed liver metastasis from human colorectal cancer cells and improved host survival compared with controls. The combination of rhLK8 and 5-fluorouracil substantially increased these survival benefits compared with either therapy alone. Histological observation showed significant induction of apoptosis among tumor-associated endothelial cells in liver metastases from rhLK8-treated mice compared with control mice. Collectively, these results suggest that the combination of rhLK8 with conventional chemotherapy may be a promising approach for the treatment of patients with life-threatening colorectal cancer liver metastases.

## Introduction

Colorectal cancer is the third most common cancer and the second leading cause of cancer death in the United States. It is estimated that in the year 2013, nearly 142,820 new cases of colorectal cancer were diagnosed and 50,830 patients died from this disease [1]. Approximately 25% of patients present with metastatic disease at diagnosis. The remaining 75% are treated surgically with cure as the objective [2], but even with complete resection the disease eventually recurs in 50% of these patients. The liver is the primary site of metastases in patients with colorectal cancer, before and after surgical removal of the primary tumor, and the formation of liver metastases constitutes a major cause of death from the disease [3]. Surgery is the primary treatment option for isolated metastases, but only 20–25% of patients are suitable for resection [4], and recurrence after surgery is frequent. Therefore, the development of a new treatment modality for this life-threatening disease is urgently needed.

Angiogenesis is the development of new capillaries from pre-existing blood vessels. Because angiogenesis is required for the expansive growth and metastasis of primary tumors, the angiogenic process is regarded as a promising target for novel cancer therapies [5], [6]. Numerous angiogenesis inhibitors, such as angiostatin and endostatin, that display significant efficacies against a variety of tumors, including metastatic colorectal cancer in pre-clinical settings, have been identified [7], [8]. The approval of bevacizumab (Avastin), a humanized monoclonal antibody against vascular endothelial growth factor, by the United States Food and Drug Administration in 2004 as a first-line therapy for metastatic colorectal cancer further validated the idea that blocking angiogenesis is an effective strategy for the treatment of human colorectal cancer.

Metastasis is a highly complex process that consists of a series of steps, including intravasation of tumor cells from the primary site into the blood or lymphatic circulation, survival of cells in the circulatory blood system, colonization of a secondary organ, initiation and maintenance of growth, and the development of new blood vessel for metastatic tumor [9], [10]. Any of these steps of metastasis may be a therapeutic target because the failure of any one step disrupts the entire metastatic cascade. However, by the time primary colorectal cancers are detected, subclinical or clinically relevant liver metastases have already occurred [11]. Accordingly, targeting the later steps of metastasis, such as the development of new vasculature, is promising because this process may not have occurred at the time of colorectal cancer diagnosis. Moreover, this step is considered less efficient than earlier steps, such as intravasation, survival in the circulation, and initial colonization at the secondary site. Biologically inefficient processes can be targeted easily because fewer cells will need to be targeted. In view of these considerations, anti-angiogenic therapy, which primarily targets the angiogenesis-dependent growth of metastases, is a clinically accessible and biologically relevant therapeutic strategy for liver metastasis.

Apolipoprotein(a) [apo(a)] is a glycoprotein component of human lipoprotein(a) and has been reported to be associated with the development of atherosclerosis and coronary heart disease [12]. Apo(a) consists of repeated kringle domains that closely resemble plasminogen kringle 4, followed by sequences that are homologous to the kringle 5 and protease domains of plasminogen [13]. The physiological role(s) of apo(a) kringle domains remain poorly understood. However, in line with the suggested role of kringles as general inhibitors of blood vessel growth [14], current evidence suggests that full-length or truncated forms of apo(a) kringles inhibit angiogenesis both *in vitro* and *in vivo* and suppress tumor growth in animal models [15], [16], [17]. We have also demonstrated that recombinant apo(a) kringle V, named rhLK8, inhibits the migration of human umbilical vein endothelial cells (HUVECs) *in vitro*, in part by interfering with the activation of focal adhesion kinases and the subsequent formation of actin stress fibers/focal adhesions [18]. rhLK8 also inhibited the neovascularization of chick chorioallantoic membranes and capillary infiltration into the Matrigel plugs *in vivo*. Recently, we demonstrated that the systemic administration of rhLK8 in combination with a chemotherapeutic agent, paclitaxel, can attenuate the growth of PC-3MM2 human prostate cancer cells in the prostate and tibia of nude mice [19]. We observed that treatment with rhLK8, especially in combination with a chemotherapeutic agent, induces apoptosis in tumor-associated endothelial cells, followed by apoptosis of the surrounding tumor cells. However, the exact biochemical mechanism by which rhLK8 induces apoptosis in endothelial cells remained unknown.

In the present study, we investigated the mechanism of rhLK8-induced apoptosis in tumor-associated endothelial cells and tested whether rhLK8 treatment, in combination with chemotherapy, suppresses colon cancer liver metastasis. We found that rhLK8 induces apoptosis in endothelial cells through the mitochondrial apoptosis pathway *in vitro.* Its interaction with glucose-regulated protein 78 (GRP78) on the endothelial cell surface may play a critical role in this process. We also demonstrated that rhLK8, especially in combination with conventional chemotherapy, significantly suppressed liver metastasis by inducing the apoptosis of tumor-associated endothelial cells *in vivo*, resulting in improved host survival in an experimental animal model of liver metastasis.

## Materials and Methods

### Expression and Purification of rhLK8 and its Derivatives

The *Saccharomyces cerevisiae* BJ3501 strain was transformed with an expression vector for *rhLK8*, which was constructed to express rhLK8 as a fusion protein with an α factor signal sequence under the control of the yeast *Gal*1 promoter and subsequently processed to be secreted into the culture medium [20]. rhLK8 proteins were purified to homogeneity from the culture supernatant of *S. cerevisiae* BJ3501 expressing rhLK8, as previously described [21]. Purified rhLK8 proteins were stored in buffer containing 100 mM NaCl and 150 mM L-glycine (pH 4.2).

The DNA fragment encoding the rhLK8 protein fused to a hemagglutinin (HA) epitope at the C-terminus (rhLK8-HA) was amplified by two cycles of polymerase chain reaction (PCR) using the following primers: rhLK8-forward (5′-TTT TTC CAT ATG GAA CAG GAC TGC ATG TTT GGG AAT GGG-3′) and HA1-reverse (5′-CAC ATC ATA AGG GTA AGA GCC CCC GCC AAA TGA AGA GGA TGC ACA GAG AGG-3′) for the first cycle using the rhLK8-expression vector as template and rhLK8-forward and HA2-reverse (5′-GGA TCC TCA AGA CCC AGA GGC ATA ATC TGG CAC ATC ATA AGG GTA AGA GCC CCC-3′) for the second cycle using the PCR product of the first cycle as template. The amplified rhLK8-HA DNA fragment was cloned into the pET-15b prokaryotic expression vector (Merck KGaA, Darmstadt, Germany), which was then used to transform *Escherichia coli* BL21 (DE3). The expression of the transgene was induced according to the manufacturer’s instructions. rhLK8-HA was expressed as a 6×His-tagged protein, and the soluble protein was affinity-purified using pET His-Tag systems (Merck KGaA) according to the manufacturer’s instructions.

### Analysis of Apoptosis by Staining with Hoechst 33452

Confluent human umbilical vein endothelial cell (HUVEC; Lonza, Walkersville, MD, USA) cultures were incubated in EBM-2 media (Lonza) supplemented with 1% FBS and various concentrations of rhLK8 (0.1–5 μM) in the presence or absence of 3 ng/ml basic fibroblast growth factor (bFGF). After an incubation period of 12 or 24 h, cells were stained with Hoechst 33452 (500 ng/ml; Sigma, St. Louis, MO, USA) for 30 min at 37°C, and apoptosis was assessed by nuclear chromatin condensation using a fluorescence microscope (Olympus BX51, Olympus, Center Valley, PA, USA) [22]. Random microscopic fields were examined for each experimental condition, and the percentage of cells that were undergoing apoptosis in each field was determined.

### Western Blotting of Apoptosis-related Proteins

Cells were lysed in Triton lysis buffer [137 mM NaCl, 2 mM EDTA, 10% glycerol, 1% Triton X-100, and 20 mM Tris-HCl (pH 8.0)] containing protease inhibitors. An aliquot of each lysate was separated by SDS-PAGE using gels polymerized from 4–20% acrylamide in Tris/Glycine buffer (Invitrogen, Carlsbad, CA, USA), and immunoblotting was performed with antibodies against procaspase-3, procaspase-9 (Cell Signaling, Beverly, MA, USA), cleaved caspase-3 and procaspase-8 (BD Biosciences, San Jose, CA, USA). Eluted samples of co-immunoprecipitation experiments were also subjected to SDS-PAGE, and the electrophoresed proteins were transferred onto nitrocellulose membranes. Each membrane was incubated with mouse anti-GRP78 antibodies (BD Biosciences; 1∶1,000) or rabbit anti-His antibodies (Santa Cruz Biotechnology, Santa Cruz, CA, USA; 1∶1,000) and then with peroxidase-conjugated anti-mouse or anti-rabbit antibodies (KPL, Gaithersburg, MD, USA; 1∶5,000).

### Fractionation of Cytosolic and Membrane-bound Proteins

Cytosolic and membrane fractions were prepared by selective plasma membrane permeabilization with digitonin, followed by membrane solubilization [23]. Briefly, cells were treated with 0.05% digitonin in isotonic buffer A [10 mM HEPES, 150 mM NaCl, 1.5 mM MgCl_2_, and 1 mM EGTA (pH 7.4)] containing protease inhibitors [1 mM 4-(2-aminoethyl) benzenesulfonyl fluoride hydrochloride, 0.8 μM aprotinin, 50 μM bestatin, 15 μM E-64, 20 μM leupeptin, and 10 μM pepstatin A] for 2 min at room temperature. The permeabilized cells were collected at 4°C. After centrifugation at 15,000×g for 10 min, the supernatant (cytosolic fraction) and the pellet (membrane fraction) were collected separately. To release membrane- and organelle-bound proteins, the pellet was further extracted with ice-cold 1% Nonidet P-40 in buffer A containing protease inhibitors for 60 min at 4°C. Both cytosolic and membrane fractions were analyzed by Western blotting using antibodies against cytochrome c (BD Biosciences).

### Construction of the Expression Vector for Glucose-regulated Protein 78 (GRP78) and Transient Transfection to HEK293 Cells

The *GRP78* gene was amplified by PCR using the following primers: forward (5′-TTT TTT GGA TCC ATG AAG CTC TCC CTG GTG GCC-3′) and reverse (5′-TTT TTT GCG GCC GCT CTA CAA CTC ATC TTT TTC TGC-3′), and then cloned into the eukaryotic expression vector pcDNA3.1-myc-his(−)b (Invitrogen). Transient transfection of HEK-293 cells with the *GRP78* expression vector was performed using lipofectamine 2000 (Invitrogen) reagents according to the manufacturer’s instructions. Twenty-four hours after the transfection, the cells were washed 3 times with phosphate-buffered saline (PBS), harvested by scraping, and centrifuged for 5 min at 500×g. The collected cells were lysed using IP buffer (150 mM NaCl, 50 mM Tris-HCl, pH 7.6) containing 1% NP-40, and cell extracts were analyzed by Western blotting.

### Co-immunoprecipitation of rhLK8-binding Proteins

HEK293 cells transfected with *GRP78* expression vectors were collected and extracted using lysis buffer (150 mM NaCl, 50 mM Tris, 1% NP-40, 1× protease inhibitor, and 1 mM phenylmethylsulfonyl fluoride, pH 7.6). The cell extracts were mixed overnight at 4°C with 10 μg of monoclonal anti-HA antibody, 2 μg of HA-tagged rhLK8, and protein G-agarose (Sigma). The immunoadsorbents were recovered by centrifugation for 5 min at 700×g, washed three times, and centrifuged (5 min at 700 × g) in IP buffer (150 mM NaCl, 50 mM Tris, 0.1% NP-40, pH 7.6). The pellets were resuspended in 30 μL of SDS-PAGE loading buffer (Sigma) and analyzed by Western blotting.

### siRNA Transfection

HUVECs were trypsinized, counted, and diluted in antibiotic-free EGM-2 medium to 2.5×10^5^ cells/ml for transfection with Dharma*FECT* 1 (Thermo Fisher Scientific, Lafayette, CO, USA), according to the manufacturer’s instructions. Briefly, HUVECs were plated into 100 mm dishes and incubated at 37°C with 5% CO_2_ overnight. One milliliter of 2 μM ON-TARGETplus SMARTpool siRNA (Thermo Fisher Scientific) against *GRP78* diluted in 1×siRNA Buffer (tube-1) and Dharma*FECT* 1 (tube-2) diluted with serum-free medium were put in separate tubes, incubated for 5 min at room temperature, mixed together, and then incubated for 20 min at room temperature. The incubated mixtures of siRNA and Dharma*FECT* 1 were diluted in 8 ml of serum-free medium and subsequently added to the 100 mm dish with HUVEC monolayers. After 24 h, the transfection medium was replaced with complete medium containing FBS and growth factors. Two days post-transfection, HUVECs were split, and on the 4th day they were harvested or replated for other experiments.

### Analysis of rhLK8 Binding to GRP78 on the HUVECs by Flow Cytometry

To measure the binding of rhLK8 to the GRP78 protein on the surface of HUVECs, rhLK8 was labeled with FITC using a FITC-labeling kit (Sigma) according to the manufacturer’s instructions. HUVECs that had been transduced with scrambled siRNA or GRP78-specific siRNA were trypsinized and neutralized by trypsin neutralizing solution (Lonza). 5×10^5^ HUVECs were stained using FITC-labeled rhLK8 or GRP78 antibodies for 1.5 h at 4°C, washed with PBS 3 times, and analyzed by flow cytometry with a FACS Caliber (BD Biosciences).

### Animal Model for Experimental Liver Metastasis

Athymic BALB/c nude mice were anesthetized by an intraperitoneal (i.p.) injection of ketamine/xylazine (Sigma). The spleen was then exteriorized through a left lateral flank incision. Approximately 3×10^5^ LS174T human colorectal carcinoma cells (American Type Culture Collection, Manassas, VA, USA) in 100 μl of Hank’s balanced salt solution were injected into the spleen parenchyma using a 27-gauge needle. The peritoneum and skin were closed in two layers with metal clips. From the day of tumor cell inoculation, mice had daily i.p. administrations of 2, 10 and 50 mg/kg rhLK8 for two weeks. In addition, control and rhLK8 (2, 10 or 50 mg/kg)-treated mice were employed in survival experiments (n = 10/each group).

To investigate the therapeutic efficacy of rhLK8 treatment in combination with a conventional chemotherapy, the mice injected with LS174T cells were randomized into four groups (n = 5 per group) and treated as follows: (1) daily i.p. administration of vehicle (100 mM NaCl, 150 mM L-Glycine, pH 4.2); (2) i.p. injection of 5-fluorouracil (5-FU; 8 mg/kg/day) for the first five days after intrasplenic injection; (3) daily i.p. injection of rhLK8 (10 mg/kg); and (4) daily i.p. administration of rhLK8 (10 mg/kg) and i.p. injection of 5-FU (8 mg/kg/day) for the first five days after intrasplenic injection. Control and rhLK8-treated mice were also used in survival experiments (n = 10 mice per group) or were euthanized by CO_2_ inhalation 2 weeks after treatment, at which time livers were collected, weighed, and analyzed to determine the surface tumor nodule numbers or were subjected to histologic and immunohistochemical examination.

### Ethics Statement

All surgical procedures were approved by the Animal Ethics Committee of the Mogam Biotechnology Research Institute or Korea Research Institute of Bioscience and Biotechnology (KRIBB-AEC-13011) and all efforts were made to minimize suffering. Care administered to the animals was in accordance with guidelines for the Care and Use of Laboratory Animals of the National Institutes of Health.

### Histology and Immunohistochemistry

To count intra-hepatic nodules, the tumor-bearing livers were dissected, fixed with 10% neutral buffered formalin overnight, embedded in paraffin, and sectioned into 4 μm slices. Sections were stained with hematoxylin and eosin (H&E) and subjected to immunohistochemical analysis. For frozen blocks, tumor specimens were dissected from mice, embedded immediately in ornithine carbamoyl transferase (OCT; Miles, Elkhart, IN, USA) compound, snap frozen in liquid nitrogen and stored at −70°C. Frozen tissues for CD31/PECAM-1 and/or terminal deoxynucleotidyl transferase-mediated dUTP nick end labeling (TUNEL) staining were prepared and fixed in cold acetone. The TUNEL assay was performed with a commercially available apoptosis detection kit (Promega, Madison, WI, USA) with some modification. Immunohistochemistry procedures were performed and all antibodies and agents for immunohistochemistry were purchased from sources as described previously [24]. Control samples exposed to secondary antibody alone showed no specific staining. The stained sections were examined under an Olympus BX51 microscope (Olympus) equipped with an Olympus DP71 12.5 megapixel digital microscope camera.

### Immunofluorescent Double Staining for CD31/PECAM-1 (Endothelial Cells) and TUNEL

The TUNEL assay was performed following CD31/PECAM-1 immunofluorescent staining as described previously [24]. Tissue samples were incubated with 300 μg/ml of the Hoechst 33342 stain for 1 min at room temperature. Propylgallate was placed on each slide and then covered with a glass cover slip (Fischer Scientific, Fair Lawn, NJ, USA). Endothelial cells were visualized with red fluorescence, and fragmented DNA (TUNEL assay) was visualized with green fluorescence. Co-localization of red and green signals produced yellow signals (apoptotic endothelial cells).

### Statistical Analysis

Data are presented as the means ± SD. Statistical significance was calculated using the Student’s *t*-test, except for the *in vivo* survival experiments, for which we used log-rank analysis of a Kaplan-Meier survival curve. A value of *p*<0.05 was considered statistically significant.

## Results

### Effects of rhLK8 on Endothelial Cell Apoptosis in vitro

**Figure 1:**
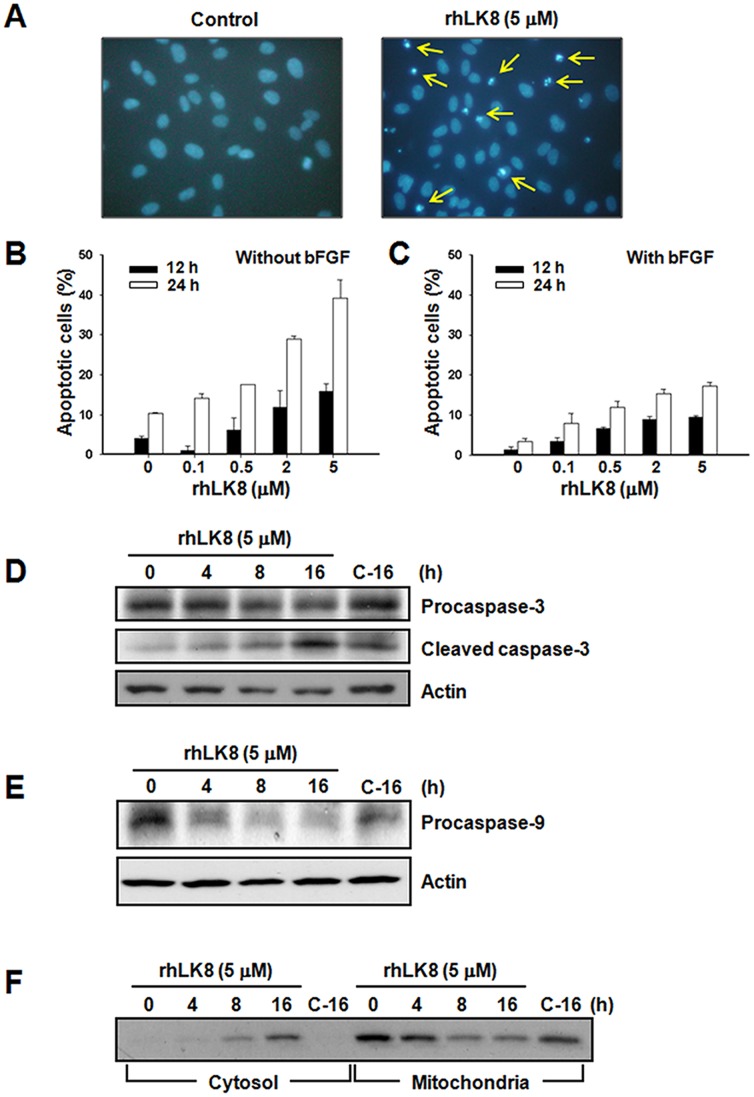
Induction of endothelial cell apoptosis by rhLK8. HUVEC monolayers were incubated in EBM-2 containing 1% FBS in the presence or absence of 3 ng/ml bFGF and treated with various concentrations of rhLK8 (0.1–5 μM) for 12 or 24 h. Endothelial apoptosis was assessed by nuclear morphology after staining with Hoechst 33452. *(A)* Representative photomicrographs of control (*left*) or rhLK8 (5 μM)-treated HUVECs (*right*) in the presence of 3 ng/ml bFGF for 12 h. Apoptotic endothelial cells are indicated by arrows. *B* and *C*, the percentage of cells undergoing apoptosis was determined in cells treated with various concentrations of rhLK8 in the absence (*B*) or presence (*C*) of bFGF after 12 (*filled bars*) or 24 h (*open bars*). Each column represents the mean ± SD. (*D–F*) HUVECs were incubated with rhLK8 (5 μM) for various time periods as indicated. Cells were then collected and lysed, and whole cell proteins were separated by SDS-PAGE. (*D*) The activation of caspase-3 was determined by Western blotting using antibodies against procaspase-3 or a 20 kDa processed form of caspase-3, as indicated. (*E*) Western blotting using antibodies against procaspase-9 was performed to determine the activation of caspase-9. Actin (*lower panel*) was used as a loading control. (*F*) Cytosolic and membrane-bound proteins were prepared as described in the “Materials and Methods” and were analyzed by Western blotting using antibodies against cytochrome c to determine the release of cytochrome c into the cytosol. Protein samples loaded in *lane C-16* were prepared from cells incubated without rhLK8 for 16 h. The immunoblots shown are representative of at least three independent experiments. (Replicates of Fig. 1D, 1E, and 1F are available in Fig. S1).

To determine the effects of rhLK8 on endothelial cell apoptosis, HUVEC monolayers were incubated in EBM-2 containing 1% FBS in the presence or absence of 3 ng/ml bFGF and treated with various concentrations of rhLK8 (0.1–5 μM) for 12 or 24 h. Apoptotic endothelial cells were identified by nuclear morphology after staining with Hoechst 33452 (Fig. 1A). Treatment with rhLK8 significantly induced the apoptosis of HUVECs in a time- and dose-dependent manner in the absence (Fig. 1B) or presence (Fig. 1C) of angiogenic factors such as 3 ng/ml bFGF.

### Caspase-3 and Caspase-9 Activation and Cytochrome C Release into the Cytosol by rhLK8

To examine the biochemical characteristics of rhLK8-induced apoptosis of HUVECs, we first tested the effects of rhLK8 on the activation of an effector caspase, caspase-3, which is a key step in apoptosis. HUVECs treated with rhLK8 (5 μM) showed decreased levels of the 32-kDa procaspase-3 but increased levels of the 20-kDa processed fragment of caspase-3 (Fig. 1D and Fig. S1A), indicating the activation of caspase-3. Cleavage of a caspase-3 substrate, poly ADP-ribose polymerase, was also detected in rhLK8-treated HUVECs (data not shown). Effector caspases are activated downstream of caspase-8 or caspase-9, which are the initiator caspases involved in signaling through two distinct apoptotic pathways, the death receptor and mitochondrial pathways, respectively [25]. To determine which pathway is responsible for rhLK8-induced endothelial cell apoptosis, we tested the effects of rhLK8 (5 μM) on the activation of caspase-8 or caspase-9. The level of procaspase-9 was significantly reduced in rhLK8-treated HUVECs compared with control cells (Fig. 1E and Fig. S1B), whereas no difference in the level of procaspase-8 was observed (data not shown), indicating that the mitochondrial pathway (also known as the intrinsic pathway) was involved. Because the mitochondrial pathway is initiated by the release of cytochrome c and other polypeptides from the mitochondrial intermembrane space into the cytosol, we examined cytochrome c release in the rhLK8-treated HUVECs. rhLK8 (5 μM) caused a time-dependent reduction in the level of cytochrome c in the mitochondrial membrane, whereas the level of cytosolic cytochrome c was concomitantly increased (Fig. 1F and Fig. S1C).

### rhLK8 Interacts with GRP78

**Figure 2:**
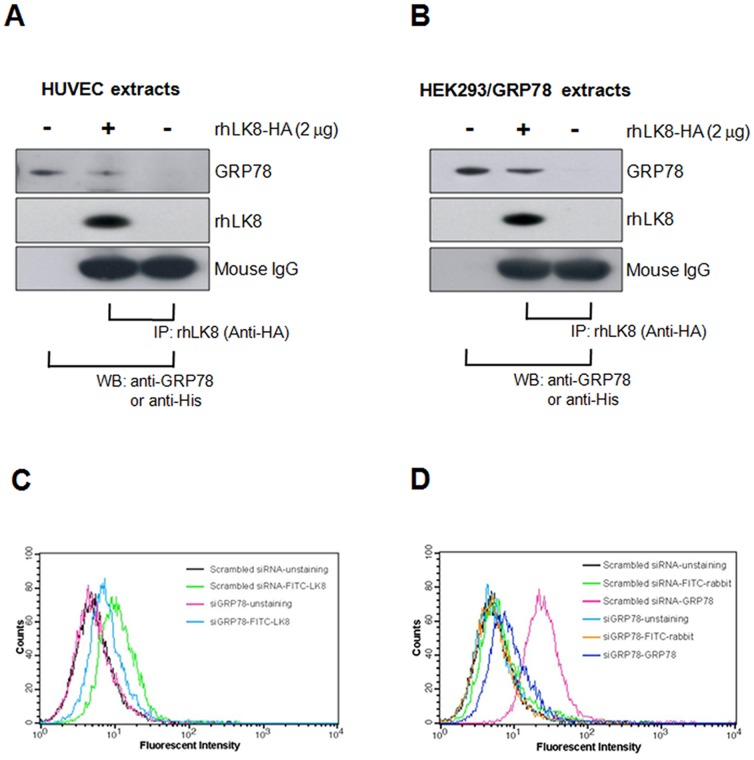
Interaction of GRP78 with rhLK8 as determined by co-immunoprecipitation and flow cytometry. For immunoprecipitation (*IP*) experiments, cell extracts of (*A*) HUVECs or (*B*) HEK293 cells expressing 6×His-tagged GRP78 protein were mixed overnight at 4°C with 10 μg of HA monoclonal antibody, 2 μg of HA-tagged rhLK8, and protein G-agarose. Eluted samples were separated by SDS-PAGE. GRP78 bound to rhLK8 was detected by Western blot (*WB*) using an anti-GRP78 monoclonal antibody or anti-His antibody. To determine the binding of rhLK8 to the GRP78 protein on the surface of HUVECs, HUVECs that had been transduced with scrambled siRNA or *GRP78-*specific siRNA were harvested and stained with (*C*) FITC-labeled rhLK8 or (*D*) anti-GRP78 antibodies and analyzed by flow cytometry. Unstained cells or cells stained with FITC-labeled secondary antibody only were used as negative control. Data are representative of two independent experiments. (Replicates of Fig. 2A and 2B are available in Fig. S2).

Recently, plasminogen kringle V (PK5) has been reported to induce the caspase-dependent apoptosis of tumor cells and endothelial cells by binding to GRP78 on the cell surface [26]. Based on the high sequence homology between PK5 and rhLK8, we tested the possibility that rhLK8 may induce the apoptosis of endothelial cells by interacting with GRP78. We mixed the extracts from HUVECs or HEK293 cells expressing His-tagged GRP78 with 10 μg of monoclonal anti-HA antibody, 2 μg of HA-tagged rhLK8, and protein G-agarose and the immunoprecipitants were immunoblotted with anti-GRP78 or anti-His antibodies. Both endogenous GRP78 in HUVECs (Fig. 2A and Fig. S2A) and His-tagged GRP78 in HEK293 cells (Fig. 2B and Fig. S2B) clearly bound to the rhLK8 added to the co-immunoprecipitation mixture. However, no GRP78 protein was detected when the co-immunoprecipitation assay was performed in the absence of HA-tagged rhLK8 protein.

### rhLK8 Binds to GRP78 Expressed on the Surface of HUVECs

To determine whether rhLK8 binds to GRP78 expressed on the surface of HUVECs, HUVECs were treated with either the specific siRNA for *GRP78* or scrambled siRNA, stained with FITC-conjugated rhLK8 or anti-GRP78 antibodies, and analyzed by flow cytometry. Both rhLK8 and GRP78 antibodies bound to the surface of HUVECs transduced with control siRNA in a dose-dependent manner, whereas the binding of rhLK8 and GRP78 antibodies was decreased in HUVECs transduced with *GRP78*-specific siRNA (Fig. 2C and 2D).

### rhLK8 induces Apoptosis of Endothelial Cells in vitro by Interacting with GRP78

**Figure 3:**
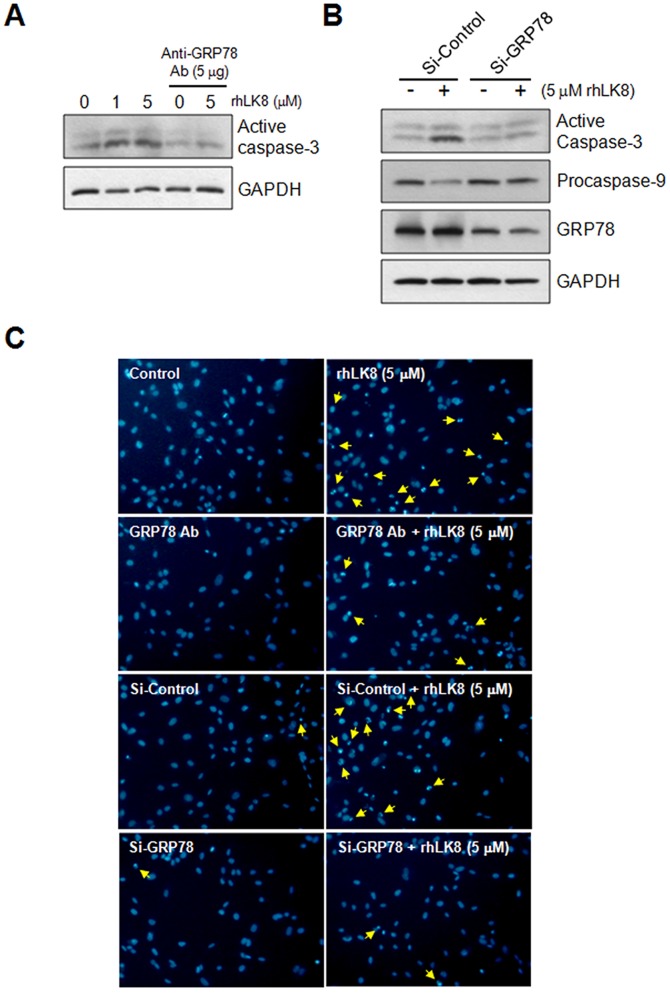
Inhibition of rhLK8-mediated endothelial cell apoptosis by *GRP78*-directed siRNA knockdown or anti-GRP78 antibodies. (*A*) To determine whether GRP78 may be involved in rhLK8-mediated endothelial cell apoptosis, HUVEC monolayers were treated with rhLK8 (1 or 5 μM) after pretreatment with 5 μg of GRP78 antibody for 30 min. Data are representative of three independent experiments. (*B*) HUVECs transfected with scrambled siRNA or *GRP78*-specific siRNA were treated with 5 μM of rhLK8. The subsequent induction of apoptosis was detected by antibodies against active caspase-3 or procaspase-9. The expression of GRP78 was detected by anti-GRP78 monoclonal antibody. GAPDH was used for loading control. Data are representative of two independent experiments. (C) HUVECs treated with GRP78 antibody or transfected with *GRP78*-specific siRNA were treated with rhLK8 (5 μM) and endothelial apoptosis was assessed by nuclear morphology after staining with Hoechst 33452. Representative photomicrographs are shown. Apoptotic endothelial cells are indicated by arrows. The magnifications are ×100. (Replicates of Fig. 3A and 3B are available in Fig. S3).

To determine whether GRP78 proteins are involved in the rhLK8-induced apoptosis of endothelial cells, HUVECs were treated with an antibody against GRP78 prior to treatment with rhLK8. Treatment with a GRP78 antibody significantly decreased the level of the active caspase-3 in HUVECs compared with control cells, indicating that GRP78 may play a role in rhLK8-mediated endothelial cell apoptosis (Fig. 3A and Fig. S3A). These data are supported further by the results showing that apoptosis in HUVECs with GRP78 siRNA knockdown was not different with or without treatment with rhLK8 (Fig. 3B and Fig. S3B–D), whereas the apoptosis of HUVECs transfected with the scrambled siRNA was significantly induced by treatment with rhLK8, as determined by the increase in active caspase-3 and the decrease in procaspase-9 (Fig. 3B and Fig. S3B–D). Consistently, GRP78 antibody treatment or *GRP78* knock-down by siRNA transfection abolished rhLK8-induced apoptosis of HUVECs at cellular level, as assessed by the number of apoptotic cells (Fig. 3C).

### Treatment with rhLK8 Suppresses the Growth of Intrasplenically Injected LS174T Cells in the Liver

**Figure 4:**
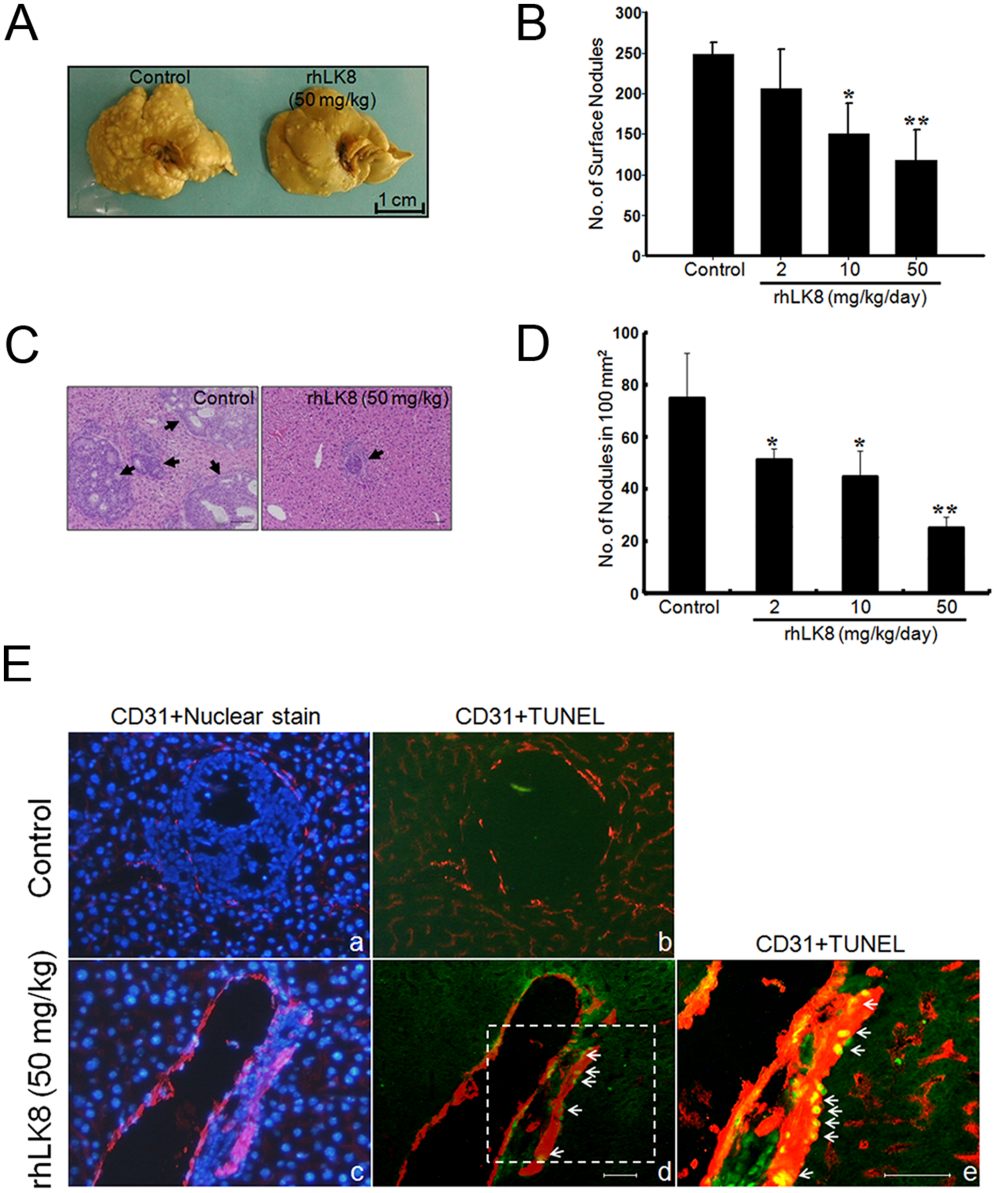
Suppression of liver metastasis by rhLK8. Approximately 3×10^5^ LS174T human colorectal carcinoma cells were injected into the spleen parenchyma of athymic BALB/c nude mice. Mice had daily i.p. administrations of rhLK8 (50, 10 or 2 mg/kg/day) for fourteen days. Mice were then sacrificed, and the livers were collected to analyze the metastasis of intrasplenically injected LS174T cells. (*A*) Representative photographs showing livers obtained from control (*left*) or rhLK8-treated (*right*) mice. (*B*) Number of surface tumor nodules in control and rhLK8-treated livers. *, *p*<0.05; **, *p*<0.01. (*C*) Sections of tumor tissues stained with H&E. *Arrows* indicate liver metastases. (*D*) The number of liver metastases per unit area (100 mm^2^) of randomly selected fields. *, *p*<0.05; **, *p*<0.01. (*E*) Immunohistochemical analyses of liver metastases of LS174T human colorectal carcinoma. Nuclei of liver specimens were stained with Hoechst 33342 (*a* and *c*), and double immunofluorescence staining was performed for CD31/PECAM-1 (red) and TUNEL (green) in control mice (*a* and *b*) or mice treated with rhLK8 (*c*, *d*, and *e*) as indicated. Apoptosis of tumor-associated endothelial cells (yellow) in the liver metastases treated with rhLK8 is indicated by arrows. Magnification, ×100. High magnification (panel *e*; ×200) of a selected region of panel (*d*), indicated by a dotted box. Bars, 100 μm. Data are representative of at least three independent experiments. (Replicates of Fig. 4A and 4B are available in Fig. S1).

As angiogenesis is critical for tumor cell metastasis and solid tumor growth, we examined the effects of rhLK8 on the liver metastasis of intrasplenically injected LS174T cells. Mice in the rhLK8 treatment group that received daily i.p. injections of 2, 10, or 50 mg/kg rhLK8 showed fewer liver metastases in a dose-dependent manner (Fig. 4A–B and Fig. S4) compared with the control group of mice. To further analyze rhLK8-mediated suppression of liver metastasis, the liver tissues were stained with H&E, and metastases were counted. There were significantly more metastasized tumors in the livers of control mice than in those of the rhLK8-treated mice (Fig. 4C). The number of liver metastases in rhLK8-treated mice was significantly lower than in control mice, and this effect was dose dependent (Fig. 4D).

### rhLK8 Induces the Apoptosis of Endothelial Cells Associated with Colon Cancer Liver Metastasis

To examine the mechanism of the inhibition of liver metastasis by rhLK8, liver tissues with colon cancer metastases from mice treated with rhLK8 (10 mg/kg/day) or vehicle for 7 days were analyzed by immunohistochemical staining for CD31/PECAM-1 and TUNEL. The CD31/TUNEL fluorescent double-labeling revealed that rhLK8 induced apoptosis of tumor-associated endothelial cells (Fig. 4E), while no substantial apoptosis was observed in control cells (Fig. 4E). Apoptosis of endothelial cells of normal liver tissue was not observed in either vehicle-treated control or rhLK8-treated groups.

### rhLK8 Improves the Survival of Mice with Colon Cancer Liver Metastases

**Figure 5:**
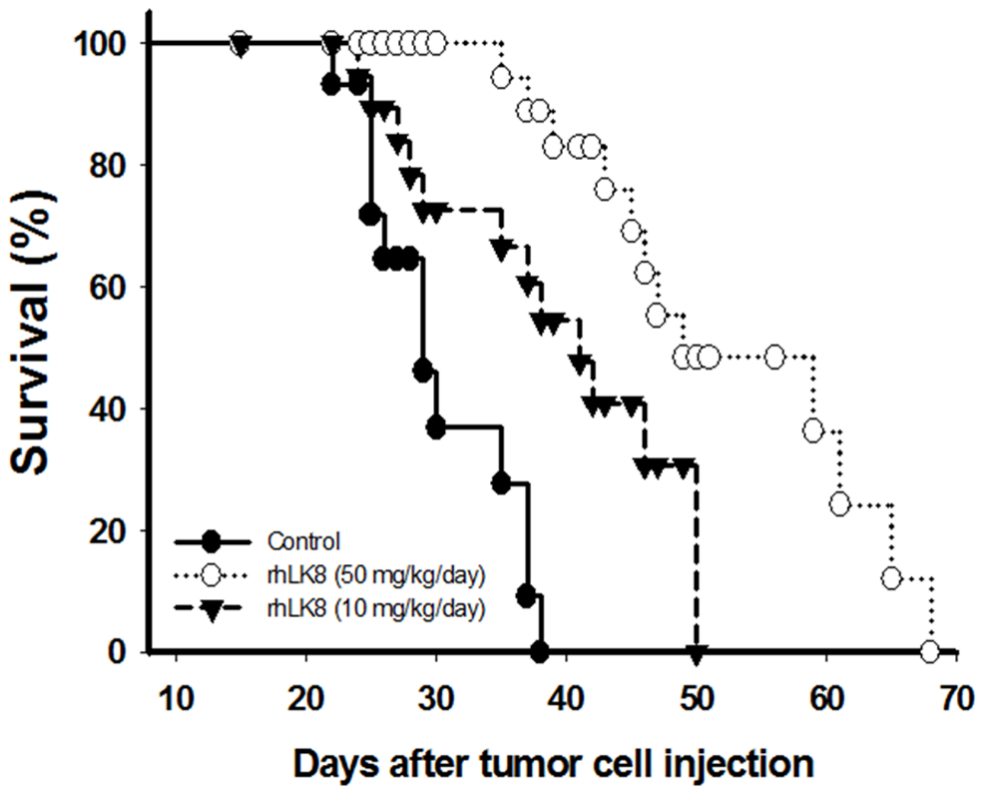
Kaplan-Meier survival curve for mice bearing liver metastases. Athymic BALB/c nude mice were injected intrasplenically with 3×10^5^ LS174T human colorectal carcinoma cells. Mice had daily i.p. administrations of 0 (•), 10 (▾) or 50 (○) mg/kg rhLK8, and the fraction of surviving mice was monitored over time. Differences in survival were statistically significant, as determined by log-rank analysis: *p*<0.005, control vs. the rhLK8-treated group (10 mg/kg); *p*<0.0001, control vs. the rhLK8-treated group (50 mg/kg). Data are representative of two independent experiments.

To assess whether the suppression of metastasis by rhLK8 translates into a survival benefit, we conducted the following survival experiment. Mice were injected intrasplenically with LS174T human colon carcinoma cells and were then treated intraperitoneally with 10 or 50 mg/kg rhLK8. The fraction of surviving animals was monitored for ∼70 days. As depicted in Fig. 5, systemic treatment with 10 or 50 mg/kg/day rhLK8 significantly improved host survival in rhLK8-treated mice compared with control mice (log-rank test; *p*<0.005 and *p*<0.0001 for mice treated with 10 or 50 mg/kg rhLK8 *vs.* control, respectively). The median survival was 29, 41 and 46 days and the overall survival was 38, 50, and 68 days in control mice and mice treated with 10 or 50 mg/kg rhLK8, respectively (Fig. 5).

### Combination Therapy of rhLK8 and 5-fluorouracil Extends the Overall Survival of Mice with Colorectal Cancer Liver Metastasis

**Figure 6:**
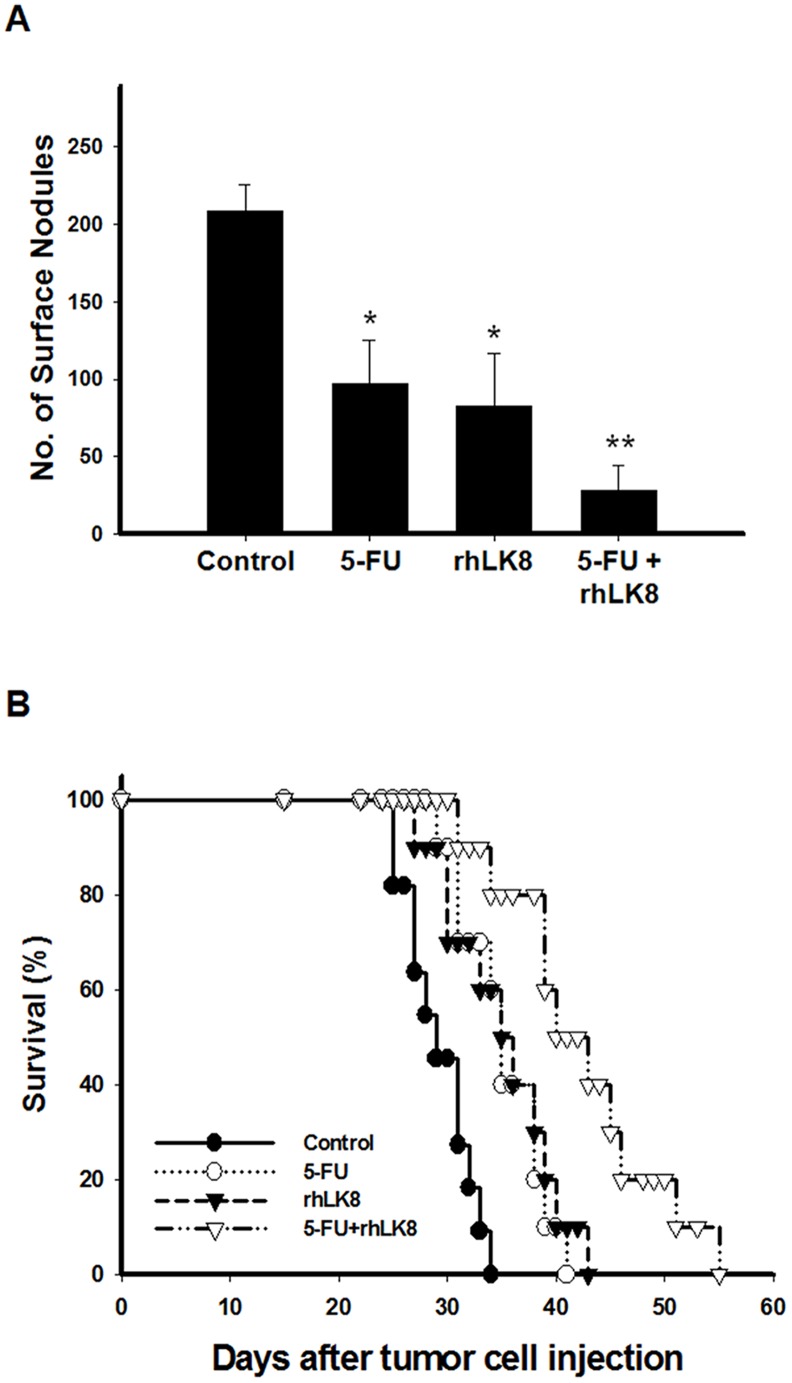
Effects of the combination of rhLK8 and conventional chemotherapy on the suppression of colon cancer liver metastasis and host survival. Mice injected intrasplenically with LS174T human colorectal carcinoma cells were administered vehicle, 5-FU, rhLK8, or 5-FU plus rhLK8, as described in “Materials and Methods”. (*A*) Mice were sacrificed, and surface nodules were counted. *, *p*<0.05 vs. control. **, *p*<0.0005 vs. control. (*B*) Kaplan-Meier survival curve of control mice (•) and mice treated with 5-FU (○), rhLK8 (▾), or 5-FU plus rhLK8 (▽). Differences in survival were statistically significant, as determined by log-rank analysis: *p*<0.005, *p*<0.001, and *p*<0.0001 vs. control mice.

Next, we determined the therapeutic effects of rhLK8 monotherapy or combination therapy with a conventional chemotherapeutic agent, 5-fluorouracil (5-FU), against LS174T cells growing in the livers of nude mice. When compared to the control group, mice treated with rhLK8 or 5-FU showed a significant reduction in the number liver metastases (Fig. 6A; *p*<0.05 vs. control group), an effect that was even more pronounced with the combination with rhLK8 and 5-FU (Fig. 6A; *p*<0.0005 vs. control group), as determined by the number of surface nodules. Moreover, host survival was significantly extended by the treatment with rhLK8 or 5-FU, and the extended host survival was improved further with the combination of rhLK8 and 5-FU, compared with the control group of mice (log-rank test; *p*<0.005, *p*<0.001, and *p*<0.0001 for mice treated with rhLK8, 5-FU, and rhLK8 plus 5-FU, respectively). The median survival was 28, 34, 34, and 39 days and the overall survival was 33, 43, 41, and 55 days after the intrasplenic injection of LS174T cells for control mice and mice treated with rhLK8, 5-FU, and rhLK8 plus 5-FU, respectively (Fig. 6B).

## Discussion

One of the main obstacles to the treatment of cancer is the heterogeneity and genetic instability of cancer cells, which leads to the development of chemoresistance [27]. For many solid tumors, despite an initial favorable response to therapy, treatment strategies developed in the past few decades have not achieved a significant increase in the median survival of patients, and improvements in prognosis are often due to better supportive care rather than to improved treatment of the cancers *per se* [28], [29]. Therefore, targeting more homogeneous and genetically stable host factors, such as the tumor vasculature, has become an attractive approach [5], [30]. In this context, anti-vascular therapy is regarded as a promising treatment strategy that could avoid drug resistance and reduce drug-related toxicities by targeting only the highly proliferative tumor-associated endothelial cells [31] rather than the relatively dormant endothelial cells in normal tissues [32].

Endothelial cell survival through the inhibition of apoptosis is thought to be an essential process during angiogenesis, whereas the induction of endothelial cell apoptosis may counteract angiogenesis [33]. Thus, angiogenesis is controlled by a balance between pro- and anti-angiogenic factors, and disturbances in this balance can trigger the cell responses required for angiogenesis [34]. Factors such as vascular endothelial growth factor and bFGF have been found to play a critical role in angiogenesis. In addition to promoting endothelial cell proliferation and migration, these pro-angiogenic factors inhibit endothelial cell apoptosis. Conversely, natural inhibitors of angiogenesis, such as angiostatin [35], have been reported to induce either the extrinsic (death receptor pathway) or intrinsic (mitochondrial pathway) apoptosis pathways in endothelial cells.

PK5 is considered the most potent angiogenesis inhibitor both *in vitro* and *in vivo* among plasminogen kringles [36], [37], and anti-angiogenic therapy with PK5 inhibits the growth of a variety of tumors [38], [39], [40], [41], [42], [43]. Though the exact mechanism of action remains to be elucidated, it has been shown that PK5 induces the apoptosis of proliferating endothelial cells [26] by interacting with a chaperone protein, the glucose-regulated protein 78 (GRP78), in the endoplasmic reticulum [26]. Binding of PK5 to GRP78 may cause the activation of caspase-7, leading to apoptotic cell death. Considering that PK5 treatment promotes the release of cytochrome c and the activation of caspase cascades including caspase-3, -6, and -7 following the mitochondrial depolarization [44], PK5 seems to induce the apoptosis of endothelial cells through the mitochondrial apoptosis pathway. In this process, regulation of the Bak/Bcl-_XL_ ratio, but not the Bax/Bcl-2 ratio, without any effects on the total amount of these proteins, has been suggested to play a critical role in the regulation of endothelial cell apoptosis [45].

In the present study, rhLK8 stimulated apoptotic turnover in endothelial cells through the mitochondria-dependent activation of caspases-9. Unlike PK5, which showed a significant sequence homology with rhLK8, our preliminary data suggest that rhLK8 increased the Bax/Bcl-2 ratio in the mitochondria but decreased the ratio in the cytosol without significantly affecting the expression of these proteins (Ahn *et al*., unpublished data). The pro-apoptotic protein Bax, which normally resides in the cytosol, translocates to mitochondria when triggered by certain stimuli. Translocated Bax has been shown to induce cytochrome c release both *in vitro* and *in vivo*, followed by caspase activation [46]. Co-immunoprecipitation and flow cytometric experiments demonstrated that rhLK8 specifically binds to GRP78 on the endothelial cell surface. GRP78 proteins appeared to be involved in the rhLK8-mediated endothelial cell apoptosis, as treatment with *GRP78* siRNA or GRP78-specific antibodies masked the rhLK8-induced apoptosis of HUVECs. However, the mechanism by which rhLK8 affects Bax translocation and how rhLK8 induces endothelial cell apoptosis through GRP78 remains unknown.

Anti-angiogenic therapy with rhLK8 may be a promising candidate for the treatment of colorectal cancer liver metastasis, as rhLK8 suppressed liver metastasis of LS174T human colorectal cancer cells in the experimental liver metastasis model in a dose-dependent manner. In line with the findings that anti-angiogenic agents may target the proliferating endothelial cells, but not the dormant endothelial cells in the normal human body, immunohistochemical analyses showed that rhLK8 induces the apoptosis of tumor-associated endothelial cells in livers from the rhLK8-treated mice but not in livers from the control group of mice or normal liver tissues. These findings may have important clinical implications because this selectivity should lead to minimal side effects even after prolonged exposure to anti-angiogenic therapy.

Most angiogenesis inhibitors confer clinical benefits primarily when combined with other conventional chemotherapies rather than when used as a monotherapy [47], as reported in the present study. With conventional chemotherapy alone, drug delivery to cancer cells may be significantly impaired by high interstitial pressure due to the inherent leakiness of the tumor vasculature. A potential explanation for the synergism of angiogenesis inhibitors and conventional chemotherapy is that anti-angiogenic therapy may initially normalize the tumor-associated vasculature, resulting in improved tissue oxygenation and increased delivery of chemotherapeutic agents [48], [49], [50].

In conclusion, these results suggest that targeting tumor angiogenesis with rhLK8 combined with a conventional chemotherapy could be a promising approach for the treatment of colorectal cancer liver metastasis.

## Supporting Information

Figure S1 **Induction of endothelial cell apoptosis by rhLK8.** HUVEC monolayers were incubated in EBM-2 containing 1% FBS in the presence of 3 ng/ml bFGF and treated with rhLK8 (5 μM) for various time periods as indicated. Cells were then collected and lysed, and whole cell proteins were separated by SDS-PAGE. (*A*) The activation of caspase-3 was determined by Western blotting using antibodies against procaspase-3 or a 20 kDa processed form of caspase-3 as indicated. (*B*) Western blotting using antibodies against procaspase-9 was performed to determine the activation of caspase-9. Actin was used as a loading control. (*C*) Cytosolic and membrane-bound proteins were prepared as described in the “Materials and Methods” and were analyzed by Western blotting using antibodies against cytochrome c to determine the release of cytochrome c into the cytosol. Protein samples loaded in *lanes C-16* or *C-24* were prepared from cells incubated without rhLK8 for 16 and 24 h, respectively. (Replicates of Fig. 1D, 1E, and 1F).
(TIF)

Figure S2 **Interaction of GRP78 with rhLK8 as determined by co-immunoprecipitation assay.** Cell extracts of (*A*) HUVECs or (*B*) HEK293 cells expressing 6×His-tagged GRP78 protein were mixed overnight at 4°C with 10 μg of HA monoclonal antibody, 2 μg of HA-tagged rhLK8, and protein G-agarose. Eluted samples were separated by SDS-PAGE. GRP78 bound to rhLK8 was detected by Western blot (*WB*) using an anti-GRP78 monoclonal antibody or anti-His antibody. (Replicates of Fig. 2A and 2B).
(TIF)

Figure S3 **Inhibition of rhLK8-mediated endothelial cell apoptosis by** ***GRP78*** **-directed siRNA knockdown or anti-GRP78 antibodies.** (*A*) To determine whether GRP78 is involved in rhLK8-mediated endothelial cell apoptosis, HUVEC monolayers were treated with rhLK8 (0–5 μM) after pretreatment with 5 μg of GRP78 antibody for 30 min. (*B*) Transfection of *GRP78*-specific siRNA decreases the expression of GRP78 proteins in HUVECs as assessed by Western blotting. (*C–D*) HUVECs transfected with scrambled siRNA or *GRP78*-specific siRNA were treated with 0–5 μM of rhLK8 as indicated. The subsequent induction of apoptosis was detected by antibodies against (*C*) active caspase-3 or (*D*) procaspase-9 as indicated. The expression of GRP78 was detected by anti-GRP78 monoclonal antibody. GAPDH was used for loading control. (Replicates of Fig. 3A and 3B).
(TIF)

Figure S4 **Suppression of liver metastasis by rhLK8.** LS174T human colorectal carcinoma cells (3×10^5^ cells) were injected into the spleen parenchyma of athymic BALB/c nude mice. Mice had daily i.p. administrations of rhLK8 (250, 50, 10, 2 or 0.4 mg/kg/day) for fourteen days. Mice were then sacrificed, and the livers were collected to analyze the metastasis of intrasplenically injected LS174T cells. (*A*) Representative photographs showing livers obtained from control or mice treated with various concentrations of rhLK8 as indicated. (*B–C*) Number of surface tumor nodules in livers from control and mice treated with various concentrations of rhLK8 as indicated. (Replicates of Fig. 4A and 4B).
(TIF)
